# The Chancellor's Difficulties and a Way out

**Published:** 1915-05-08

**Authors:** 


### HOSPITALS AND THE SPIRIT TAX.

### The Chancellor's Difficulties and a Way Out.

Until the Budget of 1909, whenever the spirit duties were raised the increases were usually at the rate of sixpence per proof gallon, the sole object being to produce a larger revenue from this source. It was hardly possible for the distiller to pass on such a small tax to the consumer, and thus the increased duty entailed no decrease in the consumption. Six years ago, however, Mr. Lloyd George tried the experiment of adding a heavy sum to the already high duties on alcohol, and in presenting his proposal to this effect he said that even if the result of the higher rate on a diminished consumption were no larger revenue it would still be conducive to the best interests of the community.

The object of the tax on spirit, in normal times, is, of course, twofold—the production of a large revenue and the placing of a limitation on the drinking of spirituous beverages; but in the past the main object has admittedly been to provide revenue. Unfortunately, however, the duty on spirit entails unintended effects; the higher the tax on whisky the higher the tax on medicinal compounds containing spirit, and consequently the higher the tax on hospitals.

Whether the large increase in the duty on spirit in 1909 had the effect of providing additional revenue, or whether it had the effect of decreasing the consumption of alcoholic beverages, can easily be proved by statistics; but from the point of view of public institutions the important question is, what effect did the increased tax have on the drug bill? Unfortunately, it had the effect of increasing the prices of a large number of drugs very substantially. Rectified spirit advanced in price seven shillings a gallon, tinctures from sixpence to tenpence per pound according to the percentage of alcohol contained in them, and the cost of over two hundred commonly prescribed drugs was raised. In the aggregate, therefore, the tax on the drug bills of hospitals and public institutions was very serious indeed. The future duty on spirit has yet to be agreed to. But were it to be doubled, the effect would be infinitely more serious. It is estimated that at one of the great London hospitals of every £250 spent on tinctures something like £225 represents the amount paid in respect of the duty on spirit. It is therefore obvious that the tax on spirit being doubled the bill for tinctures will not be very substantially short of twice the present amount, and the same applies proportionately to all other hospitals and public institutions throughout the kingdom—unless, of course, medicinal spirits are exempt from the increased taxation.

In the case of those hospitals where the use of anæsthetics prepared from rectified spirit is still continued the effect would be still more serious; but owing to the high spirit tax the amount of chloroform and ether in the manufacture of which pure spirit has been used has been reduced very substantially. Some idea of the effect of the spirit duty on the bills of those hospitals where chloroform and ether from rectified spirit are used can be obtained from the statement publicly made shortly after the Budget of 1909 was introduced that at one of the great London hospitals the anæsthetic and drug bill was taxed to the extent of £400 to £500.

When Chancellors of the Exchequer have been urged to differentiate, for taxation purposes, between potable and medicinal spirits, they have always refused on the ground that it would be difficult to provide an effective check which would ensure that the spirit was used only for strictly medicinal purposes, and to prevent frauds which might involve serious loss to the Revenue. Only a month ago, as reported in The Hospital at the time, Mr. Lloyd George stated in the House of Commons that he was afraid it would not be possible to allow hospitals to be supplied with duty-free rectified spirit for the making of tinctures under similar restrictions to those which regulate the supply of alcohol free of duty for purposes of research in laboratories and colleges. A week or two later, however, the Chancellor stated that he was considering the suggestion of giving an exemption from spirit duty to hospitals, and it is significant that the question to which Mr. Lloyd George replied suggested that it might be possible to give this special treatment to hospitals without also giving it to chemists. To distinguish between spirit used at hospitals and spirit used in the preparation of medicines for the general public might be one way of partially disposing of the difficulty, but it is questionable whether such half-measures would appeal to Mr. Lloyd George.

### Taxation and the Drug Fund.

It must be borne in mind that the Insurance Act Drug Fund is taxed to an enormous extent as a result of the duty on spirit, and in view of the Chancellor's intimate association with health insurance affairs it may reasonably be assumed that he would be just as anxious to relieve the Insurance Drug Fund from taxation as he is to help the hospitals. The Drug Fund is already in a very precarious condition, and any large increase in the spirit duty would have unfortunate consequences unless some practical means were devised for allowing a rebate in the case of tinctures and the like dispensed to insured persons.

In the case of hospitals there is a choice of methods. For instance, rectified spirit could be supplied to hospitals free of duty, and in order to prevent fraud as far as possible exact records could be kept of the uses for which the spirit was employed; or duty-paid spirit could be supplied to hospitals and a rebate allowed when records were produced showing the quantity of spirituous preparations used and dispensed. From the point of view of the Excise authorities it is probable that neither of these methods would be considered satisfactory.

An Alternative Plan.

There is another way of relieving the hospitals from this tax, but whether this is practicable or not is open to question. If a central depôt were established for manufacturing for hospitals all the preparations in the manufacture of which pure spirit is used, such a depôt could be treated as a bonded factory and warehouse, and its operations could be conducted under Excise supervision. All the hospitals drawing supplies from the depôt would no doubt be required to keep records of the purposes for which their duty-free supplies were used; and while this would entail a good deal of extra clerical work, a great economy would be effected. Central depôts could be established for large cities and for counties. Such a proposal need not necessarily revive the old question of central depôts for hospital stores generally, for the sole object would be to manufacture and supply to hospitals preparations in the manufacture of which spirit is used. This suggestion seems to be worthy of consideration. In the course of his speech in the House of Commons Mr. Lloyd George, in stating the decision of the Government with regard to the spirit tax, intimated, in answer to an interjected question, that some differentiation must be made between potable and medicinal spirits, but he gave no indication of the nature of his plans. It need hardly be said that hospital authorities have been anxiously waiting to hear what concessions it is proposed to make.

